# Skin Tone and Gender of High-Fidelity Simulation Manikins in Emergency Medicine Residency Training and their Use in Cultural Humility Training

**DOI:** 10.5811/westjem.59459

**Published:** 2023-07-12

**Authors:** Marie Anderson Wofford, Cortlyn Brown, Bernard Walston, Heidi Whiteside, Joseph Rigdon, Philip Turk

**Affiliations:** * Atrium Health, Department of Emergency Medicine, Charlotte, North Carolina; † University of North Carolina Chapel Hill School of Medicine, Chapel Hill, North Carolina; ‡ Wake Forest University School of Medicine, Department of Biostatistics and Data Science, Winston-Salem, North Carolina; § University of Mississippi Medical Center, Department of Data Science, Jackson, Mississippi

## Abstract

**Introduction:**

It is important for physicians to learn how to provide culturally sensitive care. Cultural humility is defined as a lifelong process with a goal of fixing power imbalances and creating institutional accountability through learning about another’s culture as well as performing self-exploration about one’s own beliefs, identities, and biases. One way to teach cultural humility in medicine is simulation. However, there are no peer-reviewed published studies that examine whether the skin tone or gender of the high-fidelity simulation manikins (HFSM) used by emergency medicine (EM) residency programs reflects the US population nor whether high-fidelity simulation is used to teach cultural humility. We aimed to address that gap in the literature. Our primary objective was to evaluate what proportion of EM residency programs use HFS to teach cultural humility. Our secondary objective was to evaluate whether the skin tone and gender breakdown of the EM residency program HFSM is representative of the US population.

**Methods:**

We conducted a simple random sample of 80 EM residency programs to characterize HFSM and cultural humility training. Selected programs were emailed a questionnaire. Key outcomes included HFSM skin tone and gender and whether cultural humility was taught via HFSM. We calculated point and interval estimates for the proportion of dark-, medium-, and light-toned skin and the proportion of female and male manikins. Confidence intervals were employed to test the null hypothesis that dark/medium/light skin tone was 20/20/60 and that the female/male ratio was 50/50. Both ratios were extrapolated from the US Census data.

**Results:**

Our response rate was 74% (59/80). Fifty-five of 59 EM residency programs that had manikins (0.93, 95% confidence interval [CI] 0.88–0.99) reported data on a total of 348 manikins. Thirty-nine of the 55 programs with manikins reported using HFS to teach cultural humility (0.71, 95% CI 0.60–0.82). Proportions of light-, medium-, and dark-toned manikins were 0.52 (0.43–0.62), 0.38 (0.29–0.47), and 0.10 (0.07–0.14), respectively. Proportions of male and female HFSM were 0.69 (0.64–0.76) and 0.31 (0.24–0.36), respectively. The null hypotheses that skin tone follows a 60/20/20 split and gender follows a 50/50 split were rejected, as not all confidence intervals contained these hypothesized values.

**Conclusion:**

While most EM residency programs surveyed use high-fidelity simulation to teach cultural humility, the manikins do not reflect either the skin tone or gender of the US population.

## INTRODUCTION

The current United States political climate and recent, racially charged events, coupled with evidence that minority groups often face higher rates of chronic diseases and worse medical outcomes, have highlighted the importance of diversity, equity, and inclusion (DEI) efforts within the medical field.^1^ Cultural humility is defined as a lifelong process with a goal of fixing power imbalances and creating institutional accountability through learning about another’s culture as well as performing self-exploration about one’s own beliefs, identities, and biases. The Accreditation Council for Graduate Medical Education (ACGME) requires cultural humility training in medical residency curricula. Many emergency medicine (EM) residency programs, however, have no formal DEI curricula.^2^,^3^ While lectures are the primary method of complying with ACGME teaching requirements, high-fidelity simulation (HFS) is used as well.

Although there is debate about the definition of HFS and high-fidelity simulation manikins (HFSM),^4^ for this study we used the definition provided by the simulation resource website HealthySimulation.com, which states that “high fidelity simulation is a healthcare education methodology that involves the use of sophisticated life-like manikins (sometimes called mannequins) in realistic patient environments.”^5^ Studies have shown that HFS results in improved clinical performance and provides a safe space to practice high-risk clinical scenarios and procedures.^6^,^7^ Despite this utility, there is a dearth of literature on whether HFSMs are used to teach cultural humility.

Faronda et al conducted an integrative review of simulation learning themes and found that while cultural sensitivity and competence, insight and understanding, communication, and confidence and comfort were mentioned, there were no studies that mentioned cultural humility.^8^,^9^ In addition, there is limited published research on the skin color and gender of simulation manikins. This is of particular importance in establishing a realistic environment in which the manikins reflect the patient population encountered by the trainee. In one study, 94% of manikins and simulation body parts in an advertisement brochure were white while only 6% were black.^10^ Another study reported that 68.75% of the simulation centers that responded to a survey distributed via listserves and Google Groups had manikins of color and 65.63% had body parts/task trainers of color.^11^ Of note, none of these studies focused on the use of HFSM in EM residency programs.

Our search found only one study demonstrating the use of simulation to teach cultural humility in EM training. Ward-Gaines et al used mass simulation to teach EM residents key concepts in healthcare disparities such as race/ethnicity, gender bias, stereotyping, and privilege. These residents reported increased perceived confidence of these concepts after the simulation.^2^ This study, however, was focused on standardized patients and did not include HFSM. Nor are there any peer-reviewed, published studies that explore the skin tone or gender diversity of HFSM at EM residency programs or whether these programs use HFSMs to teach cultural humility.

**Population Health Research**

*Capsule* What do we already know about this issue? *Cultural humility training is a required aspect of EM resident training. High-fidelity simulation manikins (HFSM) are a useful modality for resident education*.

What was the research question?

*Do the HFSM skin color and gender proportions reflect the US population? Do EM residency programs use HFSM to teach cultural humility?*

What was the major finding of the study?

*Light-, medium-, and dark-colored HFSM were 0.52 (95% CI: 0.43, 0.62), 0.38 (0.29, 0.47), and 0.10 (0.07, 0.14), respectively. Male and female HFSM were 0.69 (0.64, 0.76) and 0.31 (0.24, 0.36), respectively. The HFSM do not reflect the US population gender nor skin color*.

How does this improve population health?

*HFSM is a means to teach cultural humility in EM. Having HFSMs representative of the US population can potentially help trainees better care for diverse populations*.

Our first objective in this study was to evaluate what proportion of EM residency programs with HFS use HFSMs to teach cultural humility. Our second objective was to evaluate whether the skin tone and gender breakdown of the HFSMs used by these programs reflects that of the US population and, therefore, the potential patient population. We hypothesized that there would be a small percentage of EM programs that use HFSMs for cultural humility training and that the gender and skin-tone breakdown of manikins would differ from that of the overall US population.

## METHODS

### Study Design and Setting

We administered a questionnaire to a subset of EM residency programs across the country determined by simple random sampling (see Appendix 2 for details on sampling methods) from April 2021–September 2021. The study was deemed exempt by the Atrium Health Carolinas Institutional Review Board and was designed to comply with quality standards for survey reporting in medical literature.^12^

### Survey Design

As there were no questionnaires that addressed our study objectives, we collaborated with an expert survey methodologist on the creation of the survey, which we piloted to our intended audience with 25 responses. Several improvements were made through this process, including clarifying that survey questions were specific to HFSM. In addition, to reflect purchasing options, skin color was changed from ethnicity defined (eg, Black or African American) to skin tone defined (light, medium, and dark), and gender was simplified to male vs female. Response burden was decreased through skip patterns and breaks. The final questionnaire (see Appendix 1) consisted of 10 questions grouped into 1) EM residency program demographics, 2)HFSM skin tone and gender breakdown, and 3) utilization of simulation manikins to teach cultural humility. Participants generally completed the questionnaire within five minutes. The questionnaire did not include any identifying information requiring blinding.

### Data Analysis

We conducted a simple random sample of 80 of the total (220) EM residency programs in the US. Our survey was distributed via email to program directors with two follow-up phone calls for non-respondents. Several programs used a simulation center that was not affiliated with the program institution. In those cases, we distributed our survey via the methods above to the respective simulation center directors. No two programs from the simple random sample, however, used the same simulation center.

The sample size was chosen to yield confidence intervals (CI) with acceptable a priori precision for estimation (see Appendix 2). Consent was obtained electronically. We estimated proportions of EM residency programs offering scenarios with cultural humility as a primary or secondary learning goal, using survey sampling methodology described in Thompson.^13^

As we were unable to identify accurate data on the variations in skin tones in the US population, we extrapolated skin tone from the 2020 US Census race and ethnicity data.^14^ The proportion of “White alone (not Hispanic)” was classified as light skin, and the proportion of people who identified as “Asian alone,” “American Indian,” “Pacific Islander,” “two or more races,” “Hispanic or Latino,” and “Black alone” was split into approximately evenly proportioned groups of medium- and dark-skin-toned manikins (20% each of the population).

As manikin skin tone (light, medium, dark) and gender (female, male) are multinomial data clustered by simulation center, the data is likely correlated.^15^ To estimate the proportions of interest, we fit a generalized linear mixed-model with an intercept as a fixed effect and simulation center random effects. We conducted likelihood ratio tests on the random effects variance component(s) of simulation center to test for significant center-to-center variability with respect to the multinomial data. We then marginalized and refit the model and inverted the link function to recover estimates of the proportions. We used stratified block bootstrapping to obtain a set of 98.30% bootstrap percentile CIs where, importantly, resampling was done by simulation center to preserve the correlation structure among the data.^16^ We employed 98.3% CIs to preserve family wise alpha at 0.05. We tested hypotheses about representation (50/50 male/female and 60/20/20 light/medium/dark) by examining whether or not the null hypothesized values were contained by the bootstrap CIs.

## RESULTS

Survey response rate was 74% (59/80). Demographics of the responding EM residency programs are reported in Table 1. Responding programs most frequently were in the Northeast (37%), followed by the South (27%), Midwest (19%), and West (17%) and were most frequently three-year programs (71%).

Of the 59 EM residency programs that responded to the survey, 55 reported using HFSM as a part of their residency curricula (0.93, 95% CI 0.88–0.99) (Table 2); and 39 of those 55 programs with manikins reported having HFS cases where cultural humility was a primary or secondary learning objective (0.71, 95% CI 0.60–0.82). Of those 39 programs, 35 responded with the percentage (0–100) of total HFS cases using cultural humility as a primary or secondary learning objective, and the mean response was 15.9 (95% CI 11.4–20.4).

The 59 responding EM programs reported data on a total of 348 manikins. There were 5.9 manikins per EM residency program, with a range of 0–23. The generalized, linear mixed model rejected the null hypothesis of no center-to-center variability for skin tone (*P*=<0.000) and gender (*P* =<0.0001). Therefore, we proceeded with bootstrapping as described previously to estimate proportions and obtain appropriate CIs.

**Figure 1:**
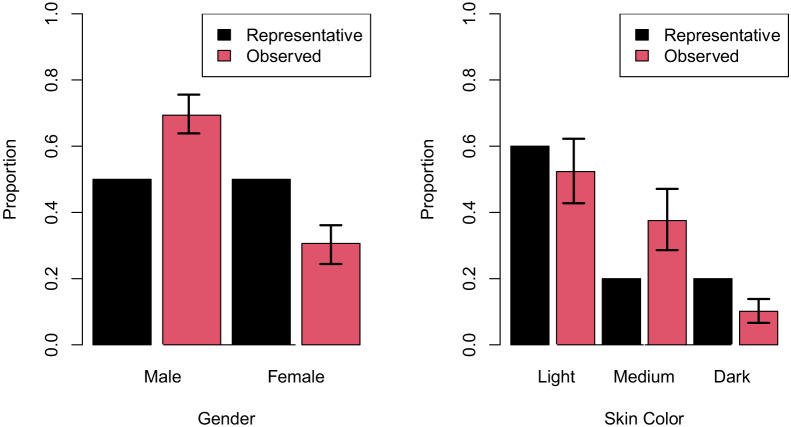
Representativeness of high-fidelity simulation manikin gender and skin tone. Error bars represent 95% confidence intervals.

Proportions of manikins with light, medium, and dark skin tones were 0.52 (0.43–0.62), 0.38 (0.29–0.47), and 0.10 (0.07–0.14), respectively. Proportions of male and female HFSM were 0.69 (0.64–0.76) and 0.31 (0.24–0.36), respectively. The null hypotheses that skin tone follows a 60/20/20 split and gender follows a 50/50 split were rejected, as not all CIs contained these hypothesized values. Results are graphically summarized in Figure 1.

When respondents were asked why their programs did not have diversity in terms of HFSM skin tone (Table 3), 20% (11/55) reported the high cost of HFSM, 22% (12/55) reported not knowing that different skin colors were available, 16% (9/55) said that at the time of purchase only one skin tone was available, and 7% (4/55) stated they did not think that having different skin colors mattered. When asked for potential reasons for the lack of diversity in terms of HFSM gender (Table 3), 20% of respondents (11/55) reported the high cost of HFSM, 13% (7/55) stated they did not think that having different genders mattered, and 11% (6/55) reported that female manikins were not available at the time of purchase. In addition, 22% (12/55) of respondents reported that some manikin genitalia can be altered to represent different genders; so, purchasers did not think it was necessary to buy manikins that, genitalia aside, represented specific genders.

## DISCUSSION

Although HFS is used in many aspects of graduate medical education, this is the first published study to our knowledge reporting the use of HFSM to teach cultural humility. Additionally, this is the first published study that reports on the skin color and gender diversity of HFSM used by EM residency programs. The findings of this study add value to the advancement of DEI initiatives in EM training.

We investigated the proportion of EM residency programs that use HFSMs to teach cultural humility as well as whether the HFSM used reflected the skin color and gender of the US population. We hypothesized that most EMRPs would not use HFSMs to teach CH and that the skin color and gender breakdown of the HFSMs would not be representative of the US population.

Our study found that while the majority of EM residency programs used HFSM to teach cultural humility, the skin tone and gender breakdown of the manikins used did not reflect the US population. More specifically, significantly, more manikins were found to be medium-colored (38%) in comparison to extrapolation of US Census data (20%). In addition, only 10% of the HFSM had dark skin tone compared to the 20% of the US population from the extrapolation of US Census data. Lastly, we found that there were significantly more male- than female-gendered HFSM. This also differs from the US Census data of an estimated 50% of the population who identify as male and female, respectively.^14^

There are numerous potential benefits to programs having diverse HFSM. First, one of the main benefits of HFS is the realistic nature of the manikins. A simulation-based learning environment should not overwhelm the learner with extraneous props and items that may create an effect of realism at the expense of cognitive burden.^4^ From our own experiences, it is very hard to stay “in character” when a phenotypically male manikin has a pink dress on to signify that it is actually female. To simplify the learning environment while ensuring realism, it is more appropriate to use the actual gender and skin color of manikins rather than adding props such as a dress.^15^

Second, as the physician workforce becomes more diverse, so is the importance of inclusivity. Faronda et al found that diversity in simulation can lead to feelings of inclusivity in trainees and aids in the creation of a more inclusive learning environment.^11^ In addition, using HFSMs to teach cultural humility can potentially act as a bridge to the exploration of topics related to disparities in care that are related, among other things, to religious backgrounds, spiritual beliefs, and the impact of one’s culture on healthcare delivery. For example, having diverse HFSM may lead to a resident becoming aware of an ethnic or gender bias that they possess. Perhaps a resident may realize that they assumed that a light-skin mannequin has high health literacy.

Ultimately, we believe that teaching cultural humility with HFSMs that reflect the actual patient population served is an important supplement to classroom lectures and will help EM trainees learn how to interact with diverse patients and provide quality healthcare through a cultural lens to patients from different values, beliefs, and behaviors.

Common themes among our survey respondents to explain the skin tone and gender disparity between HFSM and the US population were that they did not believe that “having HFSM of different skin colors or genders mattered,” “it costs too much to purchase HFSM” and that “at the time of purchase, diverse options were not available.” At present, many companies such as Laerdal Medical, CAE Health, and Gaumard Scientific make male- and female-gendered HFSM with various skin-color options. These, however, have only recently been offered or are of limited availability.^11^ For example, although simulation manikins have been available for many years, one major manufacturing company introduced a simulation mannequin available in medium and dark skin tones in 2018. In a similar vein, an African-descent upgrade kit was also made available for manikins in 2020 through this company. In terms of cost, the average price can range from $10,000–$100,000 depending on the model, type, and functionality.^17^ It is important to note, however, that for companies that offer diverse HFSM, the cost is generally the same regardless of skin color or gender.

Other programs talked about the common use of props, accessories, and vernacular to simulate the female gender and ethnicities with dark skin tones. For example, survey respondents also noted using interchangeable genitalia on a stereotypically masculine HFSM to portray a female patient. Throughout our training, we have personally seen the use of Afro wigs on manikins with light skin tones to portray a Black patient and the use of pink shirts or “stick on” female genitalia on a HFSM with a stereotypically masculine body to portray a female patient, the latter of which were commonly reported by our survey respondents. While these are a cost-effective way to “change” the gender or ethnicity of a HFSM, they may promote harmful stereotypes. In addition, these practices decrease the realism of the scenario, which undermines one of the main benefits of HFS: the ability to practice in a realistic environment.

While we acknowledge the barriers, particularly cost and availability, to obtaining diverse HFSM, we still advocate for the investment in diverse HFSM. We encourage regional and national EM and simulation societies to create grants to provide individual institutions with the funds to increase the diversity of their simulation products. In addition, programs should budget and set aside funds with a goal to increase diversity within simulation. When funds are not available, we recommend programs consider the purchase of interchangeable “headskins,” such as those by Laerdal Medical, which can serve as realistic masks to change the age or skin tone of the HFSM.^18^ While not ideal, these are a reasonably affordable option for programs interested in acquiring more diverse simulation models at a lower cost. When even these options are not available, programs should consider sharing resources with other institutions with diverse simulation products.

We also advocate for manufacturers to increase the diversity of their product offerings by creating HFSM that truly reflect various genders and ethnicities (in addition to differing body sizes/shapes and ages) rather than just “merely changing the pigment of the skin” as one respondent noted. It is not enough, however, to increase product offerings. It is important that advertising be conducted through a lens of equity as well.

In addition to ensuring diversity of simulation products, it is important to use HFSM to teach cultural humility. We were unable to find any open-access HFSM scenarios with cultural humility as a learning goal. Therefore, in addition to increasing diversity of products, we also advocate for the creation of an open-access database of HFSM scenarios focused on cultural humility that can be used by EM residency programs. One survey respondent suggested the use of “scenarios that are conducted using our translator line, if needed, and discuss language and social/cultural norms in the debrief.”

## LIMITATIONS

**Figure 2:**
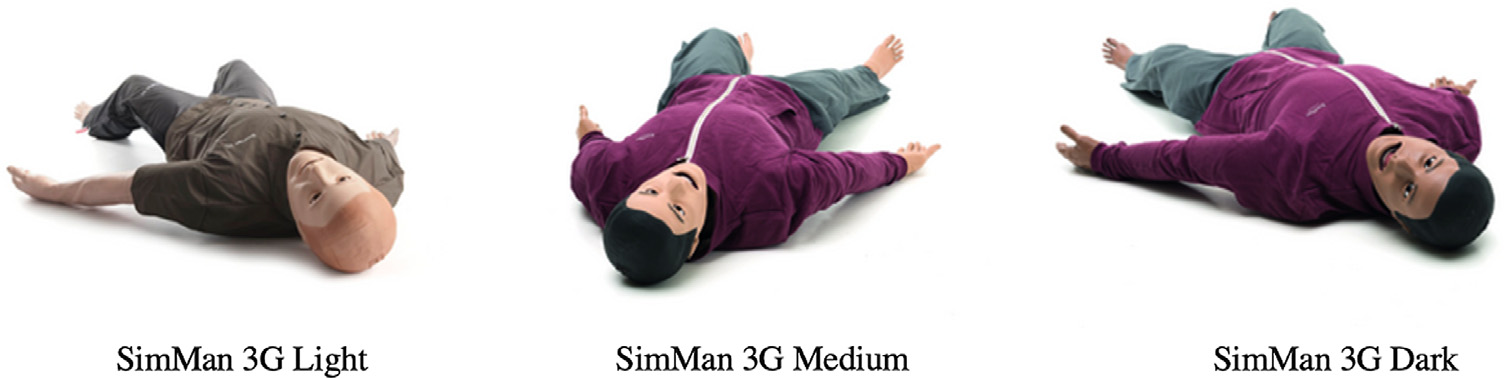
Laerdal SimMan 3G in light, medium, and dark skin tones.

There are several limitations of our study. First, the photographs used in our survey as examples of light-, medium-, and dark skin tone manikins were only from one company (Figure 2), although in our review they were consistent with product offerings from other companies. An additional limitation was our necessary extrapolation of the US Census data on ethnicity to skin color. This inherently can lead to bias but, unfortunately, was the only method to statistically categorize data. In addition, there were 21 EM residency programs that did not respond to our survey. There are many possible explanations for not responding. These EM programs may not have had HFSM (which could impose a bias on the estimate of the proportion of all EM programs that have HFSM), they may have unfortunately suffered from “diversity fatigue,”^19^ or may have just not had time to complete the survey. In addition, several respondents noted having manikins with interchangeable genitalia. To limit confusion, however, we clarified that survey responses should be based on the overall appearance of the manikin. Lastly, we also want to acknowledge that gender was reduced to a binary only to reflect the limited purchasing options for HFSMs.

## CONCLUSION

Our study found that while most EM residency programs used high-fidelity simulation manikins to teach cultural humility, the manikins used did not reflect either the skin color or gender proportions of the US population. Further studies will explore the various uses of HFS to teach cultural humility and comprehensively evaluate the effectiveness of cultural humility training in EM residency programs.

## Supplementary Information





## Figures and Tables

**Table 1:** Descriptive summary of 59 survey respondents.

Demographic information of respondent program	Respondents (n = 59)
Region	
South	16 (27%)
Northeast	22 (37%)
Midwest	11 (19%)
West	10 (17%)
Type of Program	
3-year EM	42 (71%)
4-year EM	16 (27%)
Other	1 (2%)
3–5-year EM/IM	0 (0%)
3–5-year EM/Peds	0 (0%)

*EM*, emergency medicine; *IM*, internal medicine; *Peds*, pediatrics.

**Table 2:** Quantitative analysis of high-fidelity simulation manikins (HFSM) used by emergency medicine residency programs with regard to the skin tone and gender diversity of the HFSMs and their use in cultural humility training.

Phenotypic appearance of HFSM used by respondent programs	Total Manikins (n = 348)
Manikin gender	
Male	242 (70%)
Female	106 (30%)
Manikin skin tone	
Light	183 (53%)
Medium	130 (37%)
Dark	35 (10%)
Cultural humility as a learning objective for HFS cases (n = 55)	
Yes	39 (71%)
No	16 (29%)
Percent of HFS cases with cultural humility as a primary or secondary learning objective (mean ± standard error among 35 responders)	15.9 (SE: ± 2.2)

*HFSM*, high-fidelity simulation manikins.

**Table 3:** Qualitative analysis of themes regarding why high-fidelity simulation manikins do not match the US population in terms of skin tone and gender.

Survey question	Respondents (n = 55)
If you noticed that you have very few high-fidelity simulation manikins with one skin color, why do you believe that is the case?	
Costs too much	11 (20%)
Did not know there were different skin colors available	12 (22%)
Did not think that having different skin colors mattered	4 (7%)
Other skin colors not available at time of purchase	9 (16%)
Other^1^	14 (25%)
Missing^2^	5 (9%)
If you noticed that you have very few high-fidelity simulation manikins from one gender, why do you believe that is the case?	
Costs too much	11 (20%)
Did not know there were different genders available	2 (4%)
Did not think that having different genders mattered	7 (13%)
Gender is interchangeable	12 (22%)
Female manikins not available at time of purchase	6 (11%)
Other^1^	11 (20%)
Missing^2^	6 (11%)

1 Free response that did not fit other themes.

2 No response.
