# Supplementary material for: Skin Tone and Gender of High-Fidelity Simulation Manikins in Emergency Medicine Residency Training and their Use in Cultural Humility Training
Source: West J Emerg Med. 2023 Jul 12;24(4):668–74. doi: 10.5811/westjem.59459 (PMC10393457; doi:10.5811/westjem.59459)
Supplement: Supplementary file 2 [file wjem-24-668-s002.doc]

The Skin Color and Gender of High-Fidelity Simulation Manikins in Emergency Medicine Residency Training and their use in Cultural Humility Training

**APPENDIX 2**

Because the target populations of interest in our case are finite in size, care must be taken in using standard statistical inference methods which assume sampling from an infinite size population. As a rule of thumb, the statistical underpinning for many basic methods apply reasonably well for samples taken without replacement provided the sample size is no larger than 10% of the population size.^1^

In our sample size calculation, we assumed a simple random sample without replacement of size 80 that was drawn from the population of all 220 EM residency programs in the country. We initially assumed roughly 75% of all EM programs had HFM, which would have left us with 60 EM programs (with HFM). We then assumed that roughly 10% of these EM programs might use the same simulation center. In these cases, it is likely that only two EM programs use it. These calculations led to 57 corresponding and unique simulation centers, approximately equal to 10% of the population size of all 559 simulation centers in the country that we then used to address the second study objective.

Finally, we assumed that roughly 10% of the approximately 165 EM programs with HFM also used them for CH training and fixed a confidence level of 95%. It was then shown that the margin of error of interval estimation was roughly 0.06.^2^ This precision was deemed acceptable to address this objective. Similarly, at a confidence level of 95%, it was shown that a sample size of 80 EM residency programs from all EM programs would give a margin of error for interval estimation of roughly 0.075 with respect to estimating the proportion of all EM residency programs that have HFM.

For the secondary objective, to have an idea of the precision associated with our estimation procedure in the null case, we ran an initial simulation study using a program written using the statistical software program R. For a sample of size 55 simulation centers (approximately 10% of the population size), we simulated draws from a multinomial distribution with probabilities 0.60, 0.20, and 0.20. Given limited prior information, the number of manikins for each simulation center was determined by taking a random draw from a discrete uniform distribution on 1-to-13 manikins. We than generated 1,000 resamples (by simulation center) to obtain a set of 98.3% bootstrap percentile confidence intervals. The margin of error for all three confidence intervals was roughly 0.05 for all skin colors with 95% familywise confidence. Similarly, using the same approach for sex, the margin of error for the two confidence intervals was roughly 0.05 for both sexes with 95% familywise confidence. It was felt that this level of precision was more than adequate for estimation of these proportions.

**REFERENCES**

1. Moore DS, McCabe GP, Craig BA. *Introduction to the Practice of Statistics*. Ninth edition. W.H. Freeman, Macmillan Learning; 2017.

2. Thompson SK. *Sampling*. 3rd ed. Wiley; 2012.
